# Primary Periodic Paralyses: A Review of Etiologies and Their Pathogeneses

**DOI:** 10.7759/cureus.10112

**Published:** 2020-08-29

**Authors:** Umar Farooque, Asfand Yar Cheema, Ranjeet Kumar, Gagandeep Saini, Saurabh Kataria

**Affiliations:** 1 Neurology, Dow University of Health Sciences, Karachi, PAK; 2 Medicine, Lahore Medical and Dental College, Lahore, PAK; 3 Internal Medicine, Liaquat University of Medical and Health Sciences, Jamshoro, PAK; 4 Internal Medicine, Government Medical College, Patiala, IND; 5 Neurology and Neurocritical Care, University of Missouri Health Care, Columbia, USA; 6 Neurology, West Virginia University, Morgantown, USA

**Keywords:** periodic paralysis, primary periodic paralysis, hyperkalemic periodic paralysis, hypokalemic periodic paralysis, andersen’s syndrome, congenital myasthenic syndrome, paramyotonia congenita, humans, etiology, pathogenesis

## Abstract

Periodic paralyses are a group of disorders characterized by episodes of muscle paralyses. They are mainly divided as primary (hereditary) and secondary (acquired) periodic paralyses. Primary periodic paralyses occur as a result of mutations in genes encoding subunits of muscle membrane channel proteins such as sodium, calcium, and potassium channels, resulting in impairment of their properties. Primary periodic paralyses are further classified on the basis of affected ion channels and other associated complications. Some of these periodic paralyses are hyperkalemic periodic paralysis (Na-channel mutation), hypokalemic periodic paralysis (Na- or Ca-channel mutation), Andersen’s syndrome (K-channel mutation), etc.

## Introduction and background

Periodic paralyses are a group of heterogeneous disorders characterized by flaccid paralysis and episodic attacks of muscle weakness. Periodic paralyses are mainly classified as primary and secondary periodic paralyses. Primary periodic paralyses are hereditary in which genes encoding channel protein subunits of skeletal muscle membrane are mutated, such as the muscular sodium, potassium or calcium channels, or the SCL4A1 protein [1]. Secondary periodic paralyses are, however, acquired that occur secondarily, as the name suggests, to either thyrotoxic periodic paralysis or some other renal, suprarenal, or non-renal causes, such as gastroenteritis [2]. 

The objective of this study is to review the etiologies and pathogeneses of primary periodic paralyses and discuss other related aspects.

## Review

Etiologies and pathogeneses of primary periodic paralyses

Primary periodic paralyses are caused due to mutations in genes encoding subunits of channel proteins of skeletal muscle membrane or endoplasmic reticulum like sodium, potassium, and calcium channels [1]. Following is an overview of the structure and properties of these ion channels.

Sodium Channels

The muscle membrane sodium channels, extremely crucial for muscle membrane potentials, are composed of an α‐subunit and a β‐subunit [3]. α‐Subunit consists of four homologous domains (DI-DIV) surrounding a central ion pore. Each domain comprises of six transmembrane segments (S1-S6). Sodium channels are voltage-gated highly selectively permeable channels initiating depolarization by activation (open pore) through conformational changes followed by inactivation (closed pore) blocking sodium current and causing muscle membrane to repolarize. A slight delay in inactivation of these channels can cause myotonia and muscle weakness, which is further intensified by exercise and low temperature [4]. Mutations in genes encoding sodium channels are responsible for hyperkalemic periodic paralysis, a part of hypokalemic periodic paralysis, paramyotonia congenita, and congenital myasthenic syndrome (CMS) [1].

Calcium Channels

The human calcium channels (CACNL1A3) are composed of five subunits (α1, α2, β, γ, and δ) and play an important role in excitation-contraction coupling through its association with ryanodine receptors of the endoplasmic reticulum. They act as calcium release channels and regulate intracellular calcium levels in response to changes in membrane potential. A mutation in genes that encode muscle calcium channels causes hypokalemic periodic paralysis [1].

Potassium Channels

The potassium channels are composed of four subunits arranged as mono‐ or heteromers forming a central ion-conducting pore. These channels are found in skeletal muscles, heart, and brain and are responsible for the maintenance of resting membrane potential playing an important role in repolarization and hyperpolarization required in myoblast fusion. A mutation in the potassium channel gene located on chromosome 17 results in Andersen-Tawil syndrome, causing hyperexcitability followed by inexcitability of skeletal muscle membrane [5].

Primary periodic paralyses are of various types differentiated on the basis of different gene mutations and channel proteins affected by these mutations. Several different types of primary periodic paralysis include hypokalemic, hyperkalemic, Anderson’s Syndrome, paramyotonia congenita, CMS, etc. [1].

Hyperkalemic periodic paralysis

Hyperkalemic periodic paralysis is an autosomal dominant disease in which individuals suffer from paralytic episodes in limbs and high serum potassium levels (>5 mmol/L) [6]. These episodes range from 15 minutes to 4 hours and may also affect eyes, throat, and trunk but respiratory and cardiac muscles are not known to be affected [1,6]. About half of the individuals suffering from hyperkalemic periodic paralysis report their first paralytic attack in the first decade of life usually at the age of 10 years, which becomes more frequent and severe with time up until the age of 50 years. A paralytic attack can be triggered by potassium-rich food or post-exercise rest and be intensified by low temperature and emotional stress. Most patients also have mild myotonia during each episode and some of these develop chronic progressive myopathy. Most of the older patients develop permanent muscle weakness [6].

Pathogenesis

Several different mutations may be responsible for hyperkalemic periodic paralysis. The most common mutation occurs in skeletal muscle sodium channel gene SCN4A on chromosome 17. As a consequence, fast inactivation of sodium channels is either incomplete or slowed down abnormally enhancing sodium flow making muscle fibers more incline to depolarization. This prolonged depolarization is the reason why these individuals experience muscle weakness and myotonia. High extracellular potassium level causes mild depolarization of muscle fibers that keeps mutant sodium channels in the active state causing repetitive muscle action potentials which are perceived as myotonia. If the depolarization becomes more extensive, both normal and mutant sodium channels are held in a closed state stopping all the action potentials that leads to muscle paralysis and weakness [1].

Hypokalemic periodic paralysis

Hypokalemic periodic paralysis, the most common periodic paralysis, is an autosomal dominant disorder characterized by episodes of flaccid muscle weakness lasting several hours to few days and low serum potassium level (<3.5 mmol/L) [1,7]. The first attack occurs somewhere between 2 and 30 years and might occur once in a lifetime but are usually recurring daily, weekly, monthly, etc. Paralytic attacks can be worsened by low temperature, anxiety, excessive salt ingestion, lack of exercise, consumption of glucosteroids or alcohol, and anesthetic methods. Some patients may suffer from long-lasting interictal muscle weakness [7].

Pathogenesis

Hypokalemic periodic paralysis occurs as a result of a mutation in the α‐subunit of the DHP‐receptor (CACNA1S) gene on chromosome 1 or the α‐subunit of the sodium channel (SCN4A) gene on chromosome 17 [1].

Andersen-Tawil syndrome

Andersen-Tawil Syndrome (cardiodysrhythmic periodic paralysis or potassium‐sensitive periodic paralysis) is an uncommon hereditary autosomal dominant disorder characterized by episodes of flaccid paralysis, with space of an hour to few days between each episode, along with complications like cardiac arrhythmias (like bigeminy, long QT syndrome, prolonged QUc‐interval, ventricular arrhythmias, cardiac arrest, or sudden cardiac death) and several physical deformations (like short stature, a broad forehead, hypertelorism, low‐set ears, broad‐base nose, micrognathia, cleft palate, molar hypoplasia, enamel discoloration, tapered‐curved fingers, clinodactyly, syndactyly, or scoliosis) [8]. Other uncommon associations include short palpebral fissures, persisting primary dentition, oligodontia, thin upper lip, unilateral hypoplasia or dysplasia of a kidney, dilated cardiomyopathy, heart block, semilunar valve abnormalities, bicuspid aortic valve, coarctation of the aorta, valvular pulmonary stenosis, small hands or feet, or joint laxity [9]. The signs of disease usually start to appear between 2 and 18 years of age [1].

Pathogenesis

Andersen’s syndrome occurs as a result of a mutation in gene KCNJ2 located on chromosome 17 encoding potassium channel Kir2.1 [10]. The mutation hinders flow or completely blocks the Kir2.1 channels leading to prolonged terminal phase muscle action potential. The mutation can also disturb the transport of Kir2.1 channel to muscle membrane. Prolonged depolarization, in the presence of hypokalemia, can cause cardiac arrhythmias [1]. Other complications of Andersen’s syndrome occur as a result of the tetramerization of mutant Kir2.1 allele with wild‐type Kir2.1, Kir2.2, and Kir2.3 channels [11].

Paramyotonia congenita

Paramyotonia congenita is an autosomal dominant disorder characterized by persistent non-progressive myotonia and muscle weakness mainly in eyelids, neck, and upper limb muscles, which is induced by cold temperature and further worsened by exercise [5]. These episodes of weakness may last from minutes to several hours even after removing the inducing factors. The severity or frequency is not affected by potassium intake. Only triggering factors are cold temperature and voluntary activity [1].

Pathogenesis

Paramyotonia congenita is caused by mutations in gene SCN4A located on chromosome 17 encoding skeletal muscle sodium channels. Voltage-sensitive part of domain IV is the most affected location; others including the cytoplasmic surface of transmembrane segments S2, S3, and S4. The most typical cause of paramytonia congenita is mutation segment S4 in which arginine is replaced by other amino acids nullifying its positive charge. These mutant channels are stabilized in an inactive state by low temperature, which explains the temperature sensitivity of the disease [1].

Congenital myasthenic syndrome

CMS is a heterogeneous hereditary disorder characterized by episodes of respiratory or bulbar paralysis and generalized muscle weakness. Other complications include facial weakness, ptosis, feeding difficulties, poor suck, and even arthrogryposis. Cardiac and smooth muscles are not affected. Complications start appearing at birth or soon after with varying severities [12].

Pathogenesis

CMS is caused by mutations at several genes encoding proteins of the myoneural junction. These include several subunits of the acetylcholine receptor (CHNRE, CHRNA1, CHRNB1, and CHRND), the collagenic tail subunit of the acetylcholinesterase (COLQ), choline acetyltransferase (CHAT), rapsyn (RAPSN), or sodium channel [13].

## Conclusions

This review summarizes the causes of various types of primary periodic paralyses and shines light over their complications, inducing factors, and pathogenesis at the gene level. Yet, the mechanism of their pathophysiologies is not completely understood and is needed to be studied in greater detail in future studies to completely dissect the root causes of these disorders to help mask severe complications or completely eradicate the disease.
